# Association Between Urban–Rural Geographical Location and 30-Day Survival After Out-of-Hospital Cardiac Arrest

**DOI:** 10.1016/j.acepjo.2026.100326

**Published:** 2026-02-11

**Authors:** Laura A.E. Bijman, Gareth Clegg, Nynke Halbesma

**Affiliations:** 1 Usher Institute, University of Edinburgh, Edinburgh, United Kingdom; 2 Scottish Ambulance Service, Edinburgh, United Kingdom

**Keywords:** out-of-hospital cardiac arrest, geographical area, survival

## Abstract

**Objectives:**

The aim of this study is to investigate the association between the urban–rural geographical location of out-of-hospital cardiac arrest (OHCA) and 30-day survival after OHCA in Scotland.

**Methods:**

For these analyses, data from a population-based study were utilized. In this cohort, all adult, nonemergency medical services (EMS)–witnessed patients with nontraumatic OHCA, for whom the Scottish Ambulance Service attempted resuscitation between April 1, 2011, and March 1, 2020, were included. We used the 2-fold and 6-fold urban-rural classification determined by the Scottish Government to classify cases in our cohort. We used 30-day survival after OHCA as an outcome measure. We performed univariable and multivariable logistic regression to assess the association between urban–rural geographical locations of OHCA and 30-day survival after OHCA. Furthermore, we conducted a mediation analysis to identify potential mediators.

**Results:**

This cohort included 20,378 OHCA cases. Patients living in rural areas have a lower odds of 30-day survival after OHCA compared with people living in urban areas (adjusted odds ratio, 1.19; 95% CI, 1.02 to 1.40; adjusted for age, sex, and the Scottish Index of Multiple Deprivation. Early EMS arrival time (a likely proxy for time to first defibrillation) was associated with improved 30-day survival after OHCA and was identified as a probable mediator explaining part of the association found between urban–rural geographical location of OHCA and 30-day survival after OHCA.

**Conclusion:**

Policies focusing on reducing time to first defibrillation are likely to be most effective in reducing the difference in 30-day survival after OHCA between urban and rural communities.

**The Bottom Line**

Mediating variables impacting the association between urban–rural geographical location of out-of-hospital cardiac arrest (OHCA) and 30-day survival after OHCA have not been investigated before. Early emergency medical service arrival time (a likely proxy for time to first defibrillation) was associated with improved 30-day survival after OHCA and was identified as a probable mediator explaining part of the association found between urban–rural geographical location of OHCA and 30-day survival after OHCA. Policies focusing on reducing time to first defibrillation are likely to be most effective in reducing the difference in 30-day survival after OHCA between urban and rural communities.

## 1 Introduction

### 1.1 Background

Out-of-hospital cardiac arrest (OHCA) remains an important global health burden. Each year in Europe alone around 275,000 people experience an OHCA, with only 10% eventually surviving.^1^ It is well established that there are differences in survival relating to urban–rural geographical locations of OHCA.^2^, ^3^, ^4^, ^5^ There is also evidence that paramedic exposure to OHCA (how many OHCAs a specific paramedic attended previously) might be associated with the likelihood of survival after OHCA.^2^, ^3^, ^4^ Our hypothesis is that paramedics in rural geographical locations might attend fewer OHCAs than paramedics in urban geographical locations.^6^ Furthermore, several studies reported that early arrival of the second unit of emergency medical service (EMS) significantly improved survival (survival was around 15% higher).^7^, ^8^, ^9^

### 1.2 Importance

Both the second unit of EMS arrival times and paramedic exposure in attending OHCA have not been examined before in relation to urban–rural geographical location differences in survival after OHCA. Scotland provides an excellent landscape for assessing the association between urban–rural geographical location of OHCA and 30-day survival after OHCA using the rich, linked nationwide OHCA data set.

### 1.3 Goals of This Investigation

The aim of this paper was to investigate the association between the urban–rural geographical location of OHCA and 30-day survival after OHCA. Mediating variables impacting the association between the urban–rural geographical location of OHCA and 30-day survival after OHCA have not been investigated before. This type of work is essential to identifying and prioritizing ways to optimize the system of care.

## 2 Methods

### 2.1 Study Design and Data Source

For these analyses, data from the nationwide Scottish OHCA data set were used. Data from all adult, non-EMS–witnessed patients with nontraumatic OHCA, for whom the Scottish Ambulance Service (SAS) attempted resuscitation between April 1, 2011, and March 1, 2020, were included. We excluded cases after March 1, 2020, because of the uncertain impact of the COVID-19 pandemic on the EMS response to OHCA. All details of the data collection are published elsewhere.^10^ A schematic overview of the data collection process is shown in the Figure. We have adhered to the STROBE guidelines for cohort studies.^11^

**Figure:**
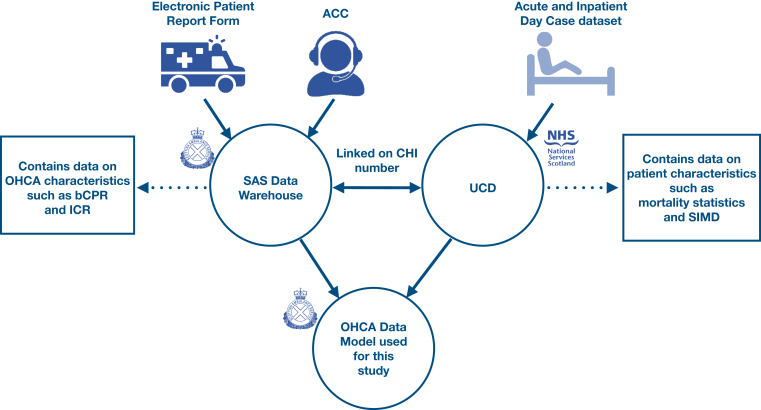
Data collection and linking process to create a cohort containing EMS-attended OHCA cases during 2011-2020 in Scotland. ACC, ambulance control center; bCPR, bystander cardiopulmonary resuscitation; CHI, community health index; EMS, emergency medical services; ICR, initial cardiac rhythm; NHS, national health service; OHCA, out-of-hospital cardiac arrest; SAS, Scottish Ambulance Service; SIMD, Scottish Index of Multiple Deprivation; UCD, unscheduled care datamart.

### 2.2 Setting

The EMS system in Scotland consists of 2 types of ambulance response vehicles that attend OHCA calls in general. Regular ambulance response vehicles consist of one ambulance paramedic and 1 ambulance technician.^12^ In some cases, an advanced paramedic attends the OHCA scene. SAS aims to “triple respond” to OHCA in cases where resuscitation is attempted—meaning at least 3 pairs of hands in attendance.^12^ The fire and rescue department and police force do not routinely respond to OHCA incidents in Scotland.^12^ The SAS uses national clinical guidelines developed by the Joint Royal Colleges Ambulance Liaison Committee (JRCALC), an organization that provides clinical oversight and expert advice to the UK’s ambulance services.^13^ Specific guidelines for termination of resuscitation in OHCA are informed by the JRCALC Recognition of Life Extinct Clinical Practice Guideline, which sets criteria for when resuscitation is unlikely to succeed.^13^

### 2.3 Measurements/Outcomes

We used the postcode of the OHCA incident location to classify cases as “urban” or “rural” using the Scottish Government Urban-Rural Classification, which was retrieved from the Rural and Environment Science and Analytical Services Division of the Scottish Government.^14^ The Scottish Government Urban–Rural Classification changes periodically as census data are updated; we used the version appropriate for the year of the record for each OHCA case. The 2-fold urban-rural classification was used in all analyses, except where indicated otherwise. The primary outcome used in all analyses was 30-day survival after OHCA as defined by death records in Scotland.

In each case, the patient’s location-based socioeconomic status was determined using the Scottish Index of Multiple Deprivation (SIMD), retrieved from the Data and Intelligence Department of the Scottish Government^15^ and linked using the patient’s home address. We classified initial cardiac rhythm (ICR) as shockable (ventricular fibrillation/ventricular tachycardia) or as nonshockable (pulseless electrical activity/asystole). EMS arrival time represented the interval from the start of the emergency call to the arrival of the first EMS unit; we used both the continuous arrival time and created a variable, in which the arrival time was dichotomized as ≤8 minutes and >8 minutes. This is the time target for OHCA ambulance response times mentioned in the SAS Local Delivery Plan.^16^ The maximum arrival time was set at 30 minutes, as longer arrival times were likely to be artifactual (eg, because the cardiac arrest occurred after initial incident coding by EMS call handlers); these were set as missing (*n* = 167, 1%). Arrival time of a second unit of EMS, ie, a “third pair of hands,” was calculated using the arrival time of the 3rd member of EMS personnel to arrive at the scene. Previous exposure of paramedics to OHCA resuscitation was identified based on the unique ID of each EMS responder. We identified how many times EMS responders had attended an OHCA incident in the previous 12 months. The OHCA exposure of all EMS responders who attended that particular OHCA incident was then combined as an arithmetic mean. The variable paramedic experience was reported as a continuous variable as well as divided into the categories ≤6 exposures in the last year, >6 and ≤11 exposures in the last year, >11 and ≤17 exposures in the last year, and >17 exposures in the last year. These categories were chosen to be able to compare the results with the most prominent study we found, looking at paramedic exposure to OHCA in relation to survival after OHCA.^17^ Location of the OHCA at home or away from home was determined by assessing whether the OHCA incident location was at the same postcode as the patient’s home address; this was used as a proxy for whether the arrest occurred at home or in a public place.

Missing data were reviewed and managed through data review. The variable EMS arrival time has 1% missing cases. The EMS arrival time missing cases are all EMS arrival times above 30 minutes and set to artifactual (see description above). None of the other variables has any missing cases.

### 2.4 Data Analysis

We reported baseline characteristics with incidence per 100,000 population, age as median interquartile range (IQR), and all other variables as percentages. For calculation of the incident, we used population numbers from Scotland’s census.^18^ *P* values were calculated using the chi-square test and Wilcoxon rank sum test. Normal distribution was tested using the Shapiro-Wilk test. The threshold we used for statistical significance was an alpha level of 0.05. We performed univariable and multivariable logistic regression to assess the association between the urban–rural geographical location of OHCA and 30-day survival after OHCA. In multivariable logistic regression, the following variables were taken into account as confounders: age, biological sex, and SIMD. Mediating variables are on the causal pathway (in contrast to confounders).^19^ We assessed the following variables as potential mediators in the association between urban–rural geographical location of OHCA and 30-day survival after OHCA: bystander cardiopulmonary resuscitation (bCPR), ICR, OHCA incident location at home or away from home, EMS arrival time, arrival time third pair of hands (second unit of EMS), and paramedic exposure to OHCA. We assessed the potential mediators by determining the difference in odds ratio (OR) between the logistic regression model adjusted for the potential mediator and the crude logistic regression model. All are adjusted for age, sex, and SIMD. We adopted the “difference-between-coefficients” approach or causal mediation, first introduced by Baron and Kenny.^20^ The cutoff point for describing a variable as a mediator was set at 10%.^21^ Furthermore, we conducted sensitivity analyses to look at the possible effect of paramedic exposure and EMS arrival time in more detail. We have performed sensitivity analyses using 2 cohorts using data from before 2015 and after 2015. We performed all analyses using R (version 4.0.5).^22^

### 2.5 Ethics Approval

This project received ethical approval from the SAS Research & Development Committee (reference number: SASRD-2020-011) and was submitted to the Health Research Authority’s decision tool for ethical input. As this is a nontransferable analysis of routinely collected data, a separate ethical review was not required.

## 3 Results

### 3.1 Demographics

In total, 20,378 non-EMS–witnessed, nontraumatic OHCA cases were included in these analyses. Table 1 shows the baseline characteristics of all cases included in the analyses, and the statistical tests that were performed. Exploratory descriptive analyses using the 6-fold urban–rural classification were undertaken and presented in Table 2.^14^ Results are consistent with analyses using the 2-fold classification.

**Table 1:** Baseline characteristics of the cohort containing EMS-attended OHCA cases during 2011-2020 in Scotland: 2-fold urban-rural classification.

Variable	Total (n = 20,378)	Urban (n = 17,056)	Rural (n = 3322)	Missing, N (%)	*P* value
**Incidence per 100,000 population/y**	42.1	42.5	40.4		
**Age, median (IQR)**	70 (57-79)	69 (56-79)	71 (60-80)		
**Sex, n (%)**					<.001^a^
Male	12,985 (63.7)	10,728 (62.9)	2257 (67.9)		
Female	7393 (36.3)	6328 (37.1)	1065 (32.1)		
**SIMD, n (%)**					<.001^a^
Most deprived, Q1	5416 (27.0)	5161 (30.9)	255 (7.8)		
Q2	4822 (24.0)	4242 (25.2)	580 (17.8)		
Q3	3854 (19.2)	2790 (16.6)	1064 (32.7)		
Q4	3216 (16.0)	2175 (12.8)	1041 (32.0)		
Least deprived Q5	2764 (13.8)	2449 (14.5)	315 (9.7)		
**Survival to 30 d, n (%)**					.070^a^
No	18,856 (92.5)	15,757 (92.4)	3099 (93.3)		
Yes	1522 (7.5)	1299 (7.6)	223 (6.7)		
**Shockable ICR, n (%)**					.972^a^
No	15,294 (75.1)	12,800 (75.0)	2494 (75.1)		
Yes	5084 (24.9)	4256 (25.0)	828 (24.9)		
**bCPR, n (%)**					<.001^a^
No	9094 (44.6)	7720 (45.3)	1374 (41.4)		
Yes	11,284 (55.4)	9336 (54.7)	1948 (58.6)		
**EMS arrival time (min), median (IQR)/n (%)**	7.6 (5.5-10.7)	7.1 (5.3-9.7)	11.5 (8.2-15.4)	177 (1)	<.001^b^
≤8	10,942 (54.2)	10,176 (60.0)	766 (23.6)		
>8	9259 (45.8)	6782 (40.0)	2477 (76.4)		
**Third pair hands arrival time (min), median (IQR)/n (%)**	13.9 (9.8-20.4)	13.0 (9.3-18.8)	20.9 (15.1-28.7)		<.001^b^
≤15	9050 (55.7)	8501 (60.7)	549 (24.4)		
>15	7210 (44.3)	5507 (39.3)	1703 (75.6)		
**Time EMS arrival (first unit) to third pair hands arrival (min), median (IQR)/n (%)**	5.2 (2.0-10.8)	5.0 (1.9-10.1)	7.8 (3.3-15.5)		<.001^b^
≤5	7887 (48.5)	7048 (50.3)	839 (37.3)		
>5	8373 (51.5)	6960 (49.7)	1413 (62.7)		
**Paramedic exposure to OHCA, median (IQR)/n (%)**	19 (12-27)	20 (13-29)	14 (8-21)		<.001^b^
**≤**6 exposures in the last year	1882 (9.2)	1204 (7.1)	678 (20.4)		
>6 and ≤11 exposures in the last year	2722 (13.4)	2055 (12.0)	667 (20.1)		
>11 and ≤17 exposures in the last year	4159 (20.4)	3408 (20.0)	751 (22.6)		
>17 exposures in the last year	11,615 (57.0)	10,389 (60.9)	1226 (36.9)		

bCPR, bystander cardiopulmonary resuscitation; EMS, emergency medical service; ICR, initial cardiac rhythm; OHCA, out-of-hospital cardiac arrest; SD, standard deviation; SIMD, Scottish Index of Multiple Deprivation; Q, quintile.

a Chi-square test.

b Wilcoxon rank sum test.

**Table 2:** Baseline characteristics of the cohort containing EMS-attended OHCA cases during 2011-2020 in Scotland: 6-fold urban-rural classification.

Variable	Total (n = 20,378)	Large urban locations (n = 7422)	Other urban locations (n = 7371)	Accessible small towns (n = 1644)	Remote small towns (n = 619)	Accessible rural locations (n = 2299)	Remote rural locations (n = 1023)	Missing, N (%)
**Incidence per 100,000 population/y**	42.1	41.1	44.5	39.2	46.5	41.4	38.4	
**Age, median (IQR)**	70 (57-79)	69 (55-79)	70 (56-79)	70 (58-79)	70 (59-80)	71 (60-80)	72 (60-81)	
**Sex, n (%)**								
Male	12,985 (63.7)	4699 (63.3)	4603 (62.4)	1037 (63.1)	389 (62.8)	1559 (67.8)	698 (68.2)	
Female	7393 (36.3)	2723 (36.7)	2768 (37.6)	607 (36.9)	230 (37.2)	740 (32.2)	325 (31.8)	
**SIMD, n (%)**								
Most deprived Q1	5416 (27.0)	2817 (38.6)	1984 (27.2)	286 (17.6)	74 (12.2)	202 (9.0)	53 (5.3)	
Q2	4822 (24.0)	1483 (20.3)	2195 (30.1)	409 (25.2)	155 (25.5)	415 (18.4)	165 (16.5)	
Q3	3854 (19.2)	919 (12.6)	1303 (17.9)	363 (22.4)	205 (33.7)	630 (28.0)	434 (43.3)	
Q4	3216 (16.0)	801 (11.0)	948 (13.0)	297 (18.3)	129 (21.2)	743 (33.0)	298 (29.7)	
Least deprived Q5	2764 (13.8)	1276 (17.5)	859 (11.8)	269 (16.5)	45 (7.4)	262 (11.6)	53 (5.2)	
**Survival to 30 d, n (%)**								
No	18,856 (92.5)	6805 (91.7)	6838 (92.8)	1543 (93.9)	571 (92.2)	2135 (92.9)	964 (94.2)	
Yes	1522 (7.5)	617 (8.3)	533 (7.2)	101 (6.1)	48 (7.8)	164 (7.1)	59 (5.8)	
**Shockable ICR, n (%)**								
No	15,294 (75.1)	5516 (74.3)	5530 (75.2)	1269 (77.2)	485 (78.4)	1722 (74.9)	772 (75.5)	
Yes	5084 (24.9)	1906 (25.7)	1841 (24.8)	375 (22.8)	134 (21.6)	577 (25.1)	251 (24.5)	
**bCPR, n (%)**								
No	9094 (44.6)	3274 (44.1)	3438 (46.6)	686 (41.7)	322 (52.0)	922 (40.1)	452 (44.2)	
Yes	11,284 (55.4)	4148 (55.9)	3933 (53.4)	958 (58.3)	297 (48.0)	1377 (59.9)	571 (55.8)	
**EMS arrival time (min), median (IQR)/n (%)**	7.6 (5.5-10.7)	7.2 (5.4-9.3)	6.9 (5.1-9.5)	9.8 (6.4-13.4)	5.6 (4.2-8.7)	11.1 (8.2-14.5)	12.6 (8.3-18.6)	177 (1)
≤8	10,942 (54.2)	4541 (61.5)	4593 (62.6)	603 (37.0)	439 (72.3)	539 (23.8)	227 (23.3)	
>8	9259 (45.8)	2842 (38.5)	2747 (37.4)	1025 (63.0)	168 (27.7)	1730 (76.2)	747 (76.7)	
**Third pair hands arrival time (min), median (IQR)/n (%)**	13.9 (9.8-20.4)	11.6 (8.8-16.1)	13.6 (9.4-19.4)	17.8 (13.6-23.8)	22.3 (13.8-29.8)	19.0 (14.1-25.5)	28.4 (21.2-37.4)	
≤15	9050 (55.7)	4512 (70.5)	3481 (57.8)	407 (33.4)	101 (28.1)	497 (29.2)	52 (9.4)	
>15	7210 (44.3)	1891 (29.5)	2546 (42.2)	811 (66.6)	259 (71.9)	1203 (70.8)	500 (90.6)	
**Time EMS arrival (first unit) to third pair hands arrival (min), median (IQR)/n (%)**	5.2 (2.0-10.8)	4.1 (1.7-8.1)	5.5 (2.0-10.8)	7.0 (3.1-13.0)	14.1 (5.3-20.7)	6.6 (2.6-13.1)	13.3 (5.5-22.7)	
≤5	7887 (48.5)	3677 (57.4)	2824 (46.9)	460 (37.8)	87 (24.2)	712 (41.9)	127 (23.0)	
>5	8373 (51.5)	2726 (42.6)	3203 (53.1)	758 (62.2)	273 (75.8)	988 (58.1)	425 (77.0)	
**Paramedic exposure to OHCA, median (IQR)/n (%)**	19 (12-27)	23 (16-33)	19 (13-26)	17 (11-24)	10 (5-15)	17 (10-24)	8 (4-13)	
**≤**6 exposures in the last year	1882 (9.2)	343 (4.6)	486 (6.6)	179 (10.9)	196 (31.7)	260 (11.3)	418 (40.9)	
>6 and ≤11 exposures in the last year	2722 (13.4)	646 (8.7)	972 (13.2)	282 (17.2)	155 (25.0)	397 (17.3)	270 (26.4)	
>11 and ≤17 exposures in the last year	4159 (20.4)	1259 (17.0)	1619 (22.0)	365 (22.2)	165 (26.7)	556 (24.2)	195 (19.1)	
>17 exposures in the last year	11,615 (57.0)	5174 (69.7)	4294 (58.2)	818 (49.7)	103 (16.6)	1086 (47.2)	140 (13.6)	

bCPR, bystander cardiopulmonary resuscitation; EMS, emergency medical service; ICR, initial cardiac rhythm; OHCA, out-of-hospital cardiac arrest; SD, standard deviation; SIMD, Scottish Index of Multiple Deprivation; Q, quintile.

### 3.2 Association Between Urban–Rural Geographical Location of OHCA and 30-Day Survival

The crude association between urban–rural geographical location of OHCA and 30-day survival after OHCA was OR 1.15 (95% CI, 0.99-1.33), favoring urban geographical locations of OHCA. The adjusted association between urban–rural geographical location of OHCA and 30-day survival after OHCA was OR 1.19 (95% CI, 1.02-1.40), favoring urban geographical locations of OHCA. We took into account the following potential confounding variables: age, sex, and SIMD. All potential confounding variables were significantly associated with the outcome. Potential mediators were not included in the adjusted analysis looking into the association between the urban–rural geographical location of OHCA and 30-day survival after OHCA. We did a separate exploratory analysis of potential mediators described below.

### 3.3 Exploratory Analyses of Potential Mediators

We identified EMS arrival time as a probable mediator in the association between the urban–rural geographical location of OHCA and 30-day survival after OHCA. Table 3 shows that only the model adjusted for EMS arrival time had more than 10% difference in OR compared with the crude model. In contrast, adjustments for ICR, bCPR, and OHCA incident location at home or away from home, arrival time of a third pair of hands (second EMS unit), and paramedic exposure to OHCA did not result in a difference of >10%. This suggests that the difference in 30-day survival after OHCA between urban and rural geographical locations of OHCA might partially be explained by the difference in EMS arrival time between urban and rural geographical locations of OHCA, which is in line with the difference in EMS arrival time presented in Table 1. Many of the associations are nonsignificant (except for bCPR and home vs away from home); because of this, the results of the mediation analysis should be interpreted with some caution.

**Table 3:** Odds ratios for 30-d survival following OHCA urban geographical locations compared to rural geographical locations in Scotland 2011-2020, estimated from different logistic regression models to assess potential mediators.

Model	OR 95% CI	% change in OR
Unadjusted	1.15 (0.99-1.33)	N/A
Adjusted for ICR	1.15 (0.99-1.35)	0.0
Adjusted for bCPR	1.17 (1.01-1.36)	1.7
Adjusted for location at home or away from home	1.22 (1.05-1.43)	6.1
Adjusted for EMS arrival time (continuous)	0.95 (0.82-1.12)	17.4
Adjusted for the third pair of hands’ arrival time (continuous)	1.07 (0.90-1.29)	7.0
Adjusted to include paramedic exposure to OHCA	1.06 (0.91-1.23)	7.8

bCPR, bystander cardiopulmonary resuscitation; EMS, emergency medical services; ICR, initial cardiac rhythm; OHCA, out-of-hospital cardiac arrest; N/A, not applicable; OR, odds ratio.

### 3.4 Additional Analyses

We have performed sensitivity analyses using 2 cohorts using data from before 2015 and after 2015; these sensitivity analyses showed comparable results. We have rerun the logistic regression analysis for both cohorts. The cohort containing cases from before 1st of March 2015 (*n* = 8541) showed an adjusted association (adjusted for sex, age, and SIMD) between urban–rural geographical location of OHCA and 30-day survival of OR 1.12 (95% CI, 0.88-1.45). The adjusted OR (adjusted for sex, age, and SIMD) for the cohort containing cases from after 1st of March 2015 and before 1st of March 2020 (*n* = 12,038) was similar (OR, 1.19 [95% CI, 0.98-1.46]). Additional analyses were performed to look at the possible effect of paramedic exposure and EMS arrival time in more detail because there is little literature reporting on paramedic exposure. To address this, we have created a table showing the best and worst-case scenarios using the paramedic exposure and EMS arrival time. Table 4 shows the percentage of 30-day survival after OHCA in the best and worst-case scenarios. The group of cases classifying as the best-case scenario shows higher 30-day survival after OHCA, independent of geographical location. The 30-day survival rate for patients with OHCA with an EMS arrival time of >8 minutes was 6.5% in urban areas compared with 6.1% in rural areas. Furthermore, in urban areas, the percentage of the first ambulance arriving after 8 minutes was 40% compared with 76.4% in rural areas. These sensitivity analyses show that EMS arrival time has a positive effect on 30-day survival after OHCA, which is in line with our other results.

**Table 4:** Percentage 30-d survival after OHCA in the best- and worst-case scenarios of urban–rural geographical locations.

Model	Urban (n = 17,056)	Rural (n = 3322)
% 30-d survival best-case scenario (≤8 min EMS arrival time and >17 paramedic exposure to OHCA) (n)	10.1 (6090)	10.0 (276)
% 30-d survival worst-case scenario (>8 min EMS arrival time and ≤6 paramedic exposure to OHCA) (n)	4.7 (438)	3.7 (471)
% 30-d survival ≤8 min EMS arrival time (n)	9.4 (10,176)	10.5 (766)
% 30-d survival >8 min EMS arrival time (n)	6.5 (6782)	6.1 (2477)
% 30-d survival >17 paramedic exposure to OHCA (n)	8.6 (10,389)	8.5 (1226)
% 30-d survival ≤6 paramedic exposure to OHCA (n)	7.3 (1204)	6.6 (678)

EMS, emergency medical services; OHCA, out-of-hospital cardiac arrest.

## 4 Limitations

Many of the results of the analyses in this study lack statistical significance or are borderline significant, meaning that results need to be interpreted with caution and further research is necessary to amplify any results found in this study. It might be argued that OHCA cases occurring in the most rural geographical locations are not always captured as “OHCAs where SAS attempted resuscitation,” if, after a longer EMS response time, the patient is clearly deceased and resuscitation is inappropriate. This could lead to survival bias, in which OHCA survival percentages overrepresented survivors in rural geographical locations, and therefore, the numbers do not reflect reality. However, because this study shows that OHCA occurring in rural geographical locations is negatively associated with 30-day survival after OHCA, any survival bias is likely to make the association stronger, not weaker. Furthermore, some of the data are nearly 15 years old. Over time, data might be recorded differently, leading to inconsistency in the data. We have performed sensitivity analyses using 2 cohorts using data from before 2015 and after 2015; these sensitivity analyses showed comparable results. We have treated an EMS arrival time of >30 minutes as anomalous; this is somewhat arbitrary. However, it was necessary to set long EMS arrival times to missing at a cutoff point. To decide this cutoff point, we have spoken to some ambulance personnel from the SAS. If EMS arrival times of >30 minutes are not anomalous, this would only strengthen our findings. Of the 175 cases in which the ambulance arrival time was longer than 30 minutes, 78 of those (44.6%) were in rural areas and 94 (53.7%) in urban areas. This strengthens our assumption that ambulance arrival times exceeding 30 minutes likely represent cases in which the initial classification by the call center was not OHCA; therefore, these ambulance arrival times are most likely not representative of the actual ambulance response time to OHCA.

Another limitation of this study is that data on lifestyle variables (such as smoking and body mass index), automated external defibrillator (AED) use, etiology of the OHCA, and comorbidities were not available to include in this study. Some of these variables might be residual confounders or mediating variables in the association between the urban–rural geographical location of OHCA and 30-day survival after OHCA. Because some of these potential confounders could not be included in this study, the confounder-adjusted association reported in this study might appear stronger or weaker than it actually is. Data on lifestyle variables are important as it is well established that populations living in rural geographical locations are less likely to have healthy lifestyle habits.^23^ In future research, if good data on lifestyle factors are available, these should be taken into account.

## 5 Discussion

This study showed that the incidence of OHCA is similar between urban geographical locations and rural geographical locations in Scotland (42.5 vs 40.4 per 100,000 population). Thirty-day survival after OHCA differed slightly between urban and rural geographical locations in Scotland (2-fold classification: 7.6% at urban geographical locations vs 6.7% at rural geographical locations; 6-fold classification: 8.3% at large urban geographical locations vs 5.8% at remote rural geographical locations).

The incidence reported in this study is in line with previously published work from Ireland^2^ and a recently published systematic review.^24^ Our study shows a crude association between urban–rural geographical location and 30-day survival after OHCA of OR 1.15 (95% CI, 0.99-1.33), favoring urban geographical locations. A study from 2023 reported a 30-day survival after OHCA crude OR 1.17 (95% CI, 1.05-1.30), favoring urban geographical locations, which is in line with our results.^25^

As expected, EMS arrival times are longer in rural geographical locations than urban geographical locations (median [IQR], 11.5 [8.2-15.4] minutes vs 7.1 [5.3-9.7] minutes). Other studies have shown the same trend.^5^ A cohort study from Canada reported a median EMS arrival time of 13.4 minutes in rural geographical locations and 7.8 minutes in urban geographical locations.^5^ Previous research shows that short EMS arrival time is an important predictor for survival after OHCA,^26^^,^^27^ with strong associations between short EMS arrival time and improved survival after OHCA reported for both rural geographical locations (adjusted OR, 4.74; 95% CI, 1.04-21.7) and urban geographical locations (adjusted OR, 3.52; 95% CI, 2.08-5.98).^4^ We identified EMS arrival time as a probable mediating variable in the association between urban–rural geographical location of OHCA and 30-day survival after OHCA. Therefore, the difference in 30-day survival after OHCA between urban and rural OHCA might partially be explained by the difference in EMS arrival time between urban and rural geographical locations. Our sensitivity analyses affirm this. The results of comparing survival rates for both urban and rural patients with OHCA with prolonged EMS arrival times (>8 minutes) support this as well. The 30-day survival rate for patients with OHCA with an EMS arrival time of >8 minutes was 6.5% (40.0% of ambulances in urban areas have an arrival time of >8 minutes) in urban areas compared with 6.1% (76.4% of ambulances in rural areas have an arrival time of >8 minutes) in rural areas.

### 5.1 Strengths

This is a population-based cohort study covering the whole of Scotland. Currently, Scotland has an ideal data environment for research into OHCA as all OHCA cases are registered with a single ambulance service and can be linked on a unique Community Health Index number^28^ to several different datasets. Scotland has geographically very rural locations as well as large urban locations; therefore, the landscape of this country suits this specific research question well. The cohort we used for all analyses is much larger compared with other studies and therefore has a lot more power and opportunities to take confounding variables into account and perform subgroup analyses. The urban-rural classification used in our cohort is well established in Scotland and is periodically updated.

The mediation analysis and additional best-case scenario analysis show that EMS arrival time appears to be the most important modifiable variable that could be targeted to improve 30-day survival after OHCA at rural geographical locations. EMS arrival time is likely to be a proxy for time to first defibrillation. These conclusions should be interpreted with some degree of caution as the associations identified are borderline statistically significant with wide CIs. Future research could look at how to effectively deploy community responders with AEDs in rural geographical locations to minimize time to first defibrillation, awaiting the first EMS unit to reach the incident location.

## Author Contributions

Laura A.E. Bijman: Conceptualization, Methodology, Formal analysis, Data Curation, Writing—Original Draft, Visualization.

Gareth Clegg: Conceptualization, Methodology, Writing—Review and Editing, Supervision.

Nynke Halbesma: Conceptualization, Methodology, Writing—Review and Editing, Supervision.

## Funding and Support

The Scottish OHCA Data Linkage Project is funded by the 10.13039/100012095Scottish Government as part of the Scottish OHCA Strategy. Laura A.E. Bijman is supported by a 10.13039/501100000274British Heart Foundation nonclinical PhD studentship (FS/19/74/34725). Nynke Halbesma is supported by a British Heart Foundation Intermediate Basic Science Research Fellowship (FS/16/36/32205).

## Conflict of Interest

All authors have affirmed they have no conflicts of interest to declare.
